# Giant Bladder Calculus in an Adult- A Persistent Problem in the Developing World: A Case Report

**DOI:** 10.5811/cpcem.2020.7.47653

**Published:** 2020-09-23

**Authors:** Ajit Kumar Vidhyarthy, Tariq Hameed, Rohit Lal, Awadh Kumar, Shivanand Sahni, Nanse Mendoza

**Affiliations:** * Darbhanga Medical College and Hospital, Department of Surgery, Darbhanga, India; † Hamdard Institute of Medical Sciences and Research, New Delhi, India; ‡ Kern Medical Center, Department of Emergency Medicine, Bakersfield, California, United States of America

**Keywords:** giant vesical calculus, gross hematuria, suprapubic cystolithotomy

## Abstract

**Introduction:**

Giant urinary bladder calculus in an adult is an uncommon entity. The number of patients with giant bladder calculi has decreased over recent years owing to wider availability of healthcare and better diagnostic modalities.

**Case Report:**

We present a case of a young adult without any history of recurrent urinary tract infections or bladder outlet obstruction with giant vesical calculus who presented to the emergency department with gross hematuria, abdominal pain, and dysuria. Investigations revealed a large calculus in the urinary bladder, and suprapubic cystolithotomy was performed. A large stone of 6.5×6×5.5 centimeters, weighing 125 grams, was removed. On follow-up, the patient was free of any symptoms and cystoscopy was normal.

**Conclusion:**

Urinary outflow obstruction must be ruled out in all patients with giant vesical calculus. Patients without any predisposing condition should be treated as a separate entity and evaluated accordingly. Multiple surgical treatment modalities are available for bladder calculus patients. Treatment is personalised as per size of stone, number of stones, and associated comorbidities.

## INTRODUCTION

Urinary bladder calculi comprise approximately 5% of all cases of urolithiasis.^1^ A urinary bladder calculus more than100 gram (gm) in weight is classically labelled as giant vesical calculus.^2^ Fewer than 100 cases have been reported in the literature with weight more than 100 gm and almost all of them had bladder outlet obstruction.^3^ Not many cases of giant vesical calculus are reported in the modern era because of widespread access to healthcare facilities including radiograph and ultrasonography. Bladder calculi have a varied presentation ranging from completely asymptomatic to dysuria, lower abdominal pain, gross hematuria, and retention of urine.^4^ Here we report a case of giant vesical calculus in an otherwise healthy adult male who presented to the emergency department (ED) with gross hematuria, lower abdominal pain, and dysuria.

## CASE REPORT

A 34-year-old Indian male presented to the ED with complaints of gross hematuria, severe dysuria, and pain in the lower abdomen for two days. He reported a history of 4–5 episodes of increased frequency of micturition associated with poor urinary stream and dull aching pain in his lower abdomen during the prior three years. He also reported passing blood in his urine occasionally in the preceding month, which was towards the end of the act of micturition. The patient was non-diabetic and denied history of tobacco or alcohol abuse. On physical examination he was afebrile and there was tenderness and fullness in the hypogastrium. Prostate was normal on digital rectal examination. A three-way Foley catheter was introduced and the bladder irrigated with normal saline after taking urine samples for routine and microscopic examination.

**Image 1:**
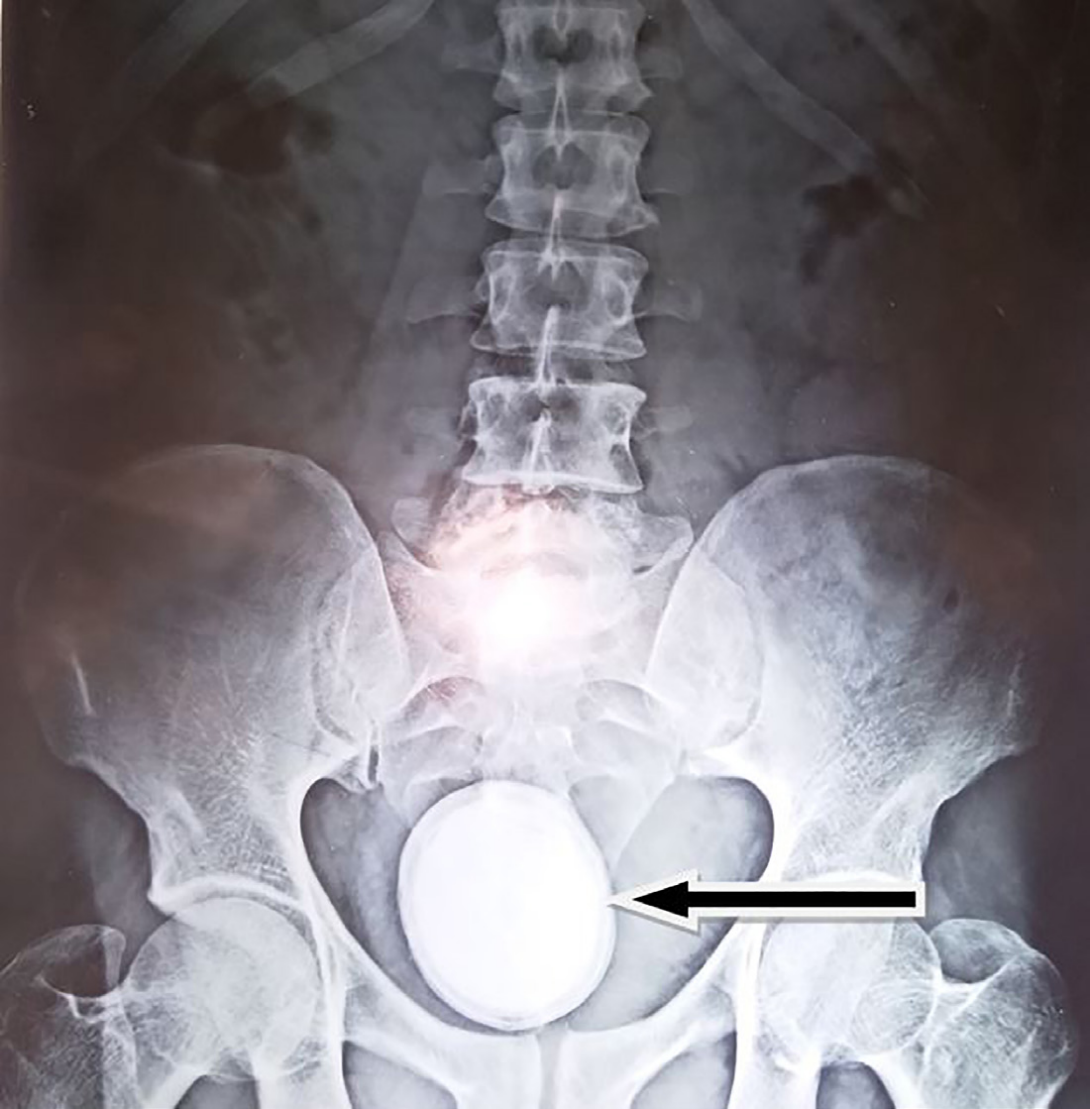
Radiograph of kidney, ureters and bladder showing a giant radiodensity overlying the bladder.

**Image 2:**
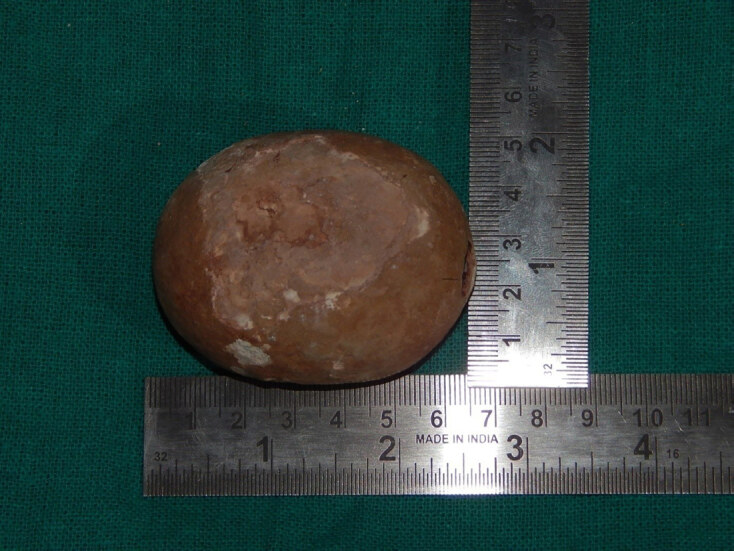
Giant vesical calculus measuring 6.5 × 6 × 5 centimeters.

Urine lab work showed the presence of red blood cells (RBC) more than 200 per high power field (HPF) (reference range 4 RBC/HPF), and pus cells were 30/HPF. Urine culture showed *Escherichia coli* sensitive to *amikacin*. His total leucocyte count was 13,800 per microliter (μl) (reference range 4000–11000/μl) with neutrophils accounting for 84% in differential leucocyte count. His blood urea, serum creatinine, and blood glucose levels were in normal range. Ultrasonography suggested a large vesical calculus with bilateral normal kidneys. Radiography of kidney-ureter-bladder revealed a radio-opaque shadow in the pelvic region measuring approximately 6×5 cm in size (Image 1). Antibiotics were started to control urinary tract infection, and open cystolithotomy was performed. A large calculus (6.5×6×5 cm, and weighing 125 gm) was removed (Image 2). Postoperative period was uneventful. The patient was discharged on postoperative day 3, and the catheter was removed at postoperative day 14. The patient had no further episodes of hematuria, and his lower urinary tract symptoms were also relieved. At six weeks post-surgery, follow-up cystoscopy was normal without any residual stone, bladder outlet obstruction, or cystitis/urethritis.

CPC-EM Capsule

What do we already know about this clinical entity?

Urinary bladder calculus is an uncommon problem. This is an important subset of all types

*of urolithiasis*.

What makes this presentation of disease reportable?

*Due to better diagnostics, vesical calculi are found early which makes it unusual to have a young adult with giant vesical calculus coming to emergency department*.

What is the major learning point?

*Giant vesical calculus may present without infravesical obstruction. In such cases etiology of stone formation should be thoroughly investigated*.

How might this improve emergency medicine practice?

*As demonstrated in this case, giant vesical calculus must be considered in young adults presenting to emergency departments*.

## DISCUSSION

Urinary tract stones have continued to be a cause of concern for humans since time immemorial, and symptoms of bladder calculus were described by Hippocrates.^5^ Prevalence rate of urinary tract stones in developed countries is between 4–20%, while in Asia it is estimated to be between 1–19.1%.^6^,^7^ Bladder calculi are usually secondary to calculi in the kidney or ureter, and it is rare to find primary bladder calculus in healthy adults. Bladder calculi constitute approximately 5% of all urinary tract stones.^1^ Bladder stones in adults are composed of uric acid in almost 50% of the cases without having features of gout or hyperuricemia.^8^

All factors causing urinary stasis, such as benign prostatic hyperplasia, neurogenic bladder, urethral strictures, and recurrent urinary tract infections, lead to formation of stones in urinary bladder.^9^ Foreign bodies such as stents and catheter act as niduses for stone formation.^1^ A giant bladder stone was reported in a patient who had undergone augmentation cystoplasty.^9^ It is unusual to have giant bladder stone without any such predisposing condition and this needs further discussion and investigations. Giant bladder calculi without any known predisposing factor should be considered as a separate entity, and further evaluation regarding etiology and treatment is required.

Bladder stones have varied clinical presentations ranging from completely asymptomatic to acute retention of urine. A young male with giant vesical calculus was having defecatory problems, and his symptoms resolved on removal of stone.^10^ Giant vesical calculus has also been reported with bilateral hydronephrosis.^10^ Patients have also presented with pollakiuria and hematuria.^3^ A case has been described in the literature where a giant vesical calculus presented as acute renal failure.^11^ In children, bladder stones may present as priapism and enuresis.^12^

Treatment of bladder stones has undergone a sea change in last few centuries. The first documented suprapubic lithotomy was performed by Pierre Franco in 1561.^4^ Bigelow in 1874 advanced bladder stone surgery by designing a larger lithotrite with which he performed litholapaxy.^4^ Recently advanced endourological procedures have replaced an open approach to urinary tract stones but an open surgical approach is still used if stones have atypical presentation, are large or multiple in number, or if advanced facilities are not available.^13^,^14^ Endourological procedures such as pneumatic lithotripsy are equally effective and safe with considerably lower morbidity.^15^ Percutaneous cystolithotripsy has a greater than 90% stone-free rate with the added benefit of no increased risk of developing urethral stricture as no endoscopic sheath is passed per urethra.^16^,^17^ Stone clearance rate and success of lithotripsy also depends on size and Hounsfield unit of stones.^18^ Extracorporeal shock wave lithotripsy is better for high-risk surgical patients or those having smaller stones.^16^

## CONCLUSION

Giant urinary bladder stones are rare and have different ways of presentation. Finding the cause of stone formation is as important as diagnosis itself. Any pathology causing bladder outlet obstruction must be diagnosed and treated to prevent recurrence. A young male without any infravesical obstruction or recurrent urinary tract infections presenting with giant vesical calculus is an uncommon presentation. Such giant stones should be treated as a separate entity, and further research must be conducted to find the etiology of stone formation in such cases. Prompt diagnosis, early intervention, and follow-up are paramount to having a good prognosis. Treatment modality for each patient differs with respect to the size and number of stones and associated comorbidities.
